# Medial prefrontal cortex dopamine controls the persistent storage of aversive memories

**DOI:** 10.3389/fnbeh.2014.00408

**Published:** 2014-11-26

**Authors:** María C. Gonzalez, Cecilia P. Kramar, Micol Tomaiuolo, Cynthia Katche, Noelia Weisstaub, Martín Cammarota, Jorge H. Medina

**Affiliations:** ^1^Laboratorio de Memoria, Facultad de Medicina, Instituto de Biología Celular y Neurociencia, Universidad de Buenos Aires-CONICETBuenos Aires, Argentina; ^2^Grupo de Neurociencias de Sistemas, IFIBIO Houssay, Departamento de Fisiología, Facultad de Medicina, Universidad de Buenos AiresBuenos Aires, Argentina; ^3^Memory Research Laboratory, Brain Institute, Federal University of Rio Grande do Norte (UFRN)Natal, Brazil; ^4^Departamento de Fisiología, Facultad de Medicina, Universidad de Buenos AiresBuenos Aires, Argentina

**Keywords:** inhibitory avoidance, conditioned taste aversion, D1/D5 receptor, VTA, system consolidation

## Abstract

Medial prefrontal cortex (mPFC) is essential for initial memory processing and expression but its involvement in persistent memory storage has seldom been studied. Using the hippocampus dependent inhibitory avoidance learning task and the hippocampus-independent conditioned taste aversion paradigm together with specific dopamine receptor agonists and antagonists we found that persistence but not formation of long-term aversive memories requires dopamine D1/D5 receptors activation in mPFC immediately after training and, depending on the task, between 6 and 12 h later. Our results indicate that besides its well-known participation in retrieval and early consolidation, mPFC also modulates the endurance of long-lasting aversive memories regardless of whether formation of the aversive mnemonic trace requires the participation of the hippocampus.

## Introduction

Medial prefrontal cortex (mPFC) plays a critical role in remote memory retrieval as well as in the consolidation and recall of recent memories (Runyan et al., 2004; Frankland and Bontempi, 2005; Gonzalez et al., 2013). mPFC dopamine (DA) signaling has been involved in cognitive, emotional and motivational processes (Seamans et al., 1998; Pezze et al., 2003; Laviolette et al., 2005; Lauzon et al., 2009). The ventral tegmental area (VTA) is the primary source of DA afferents to the mPFC (Lammel et al., 2012). These projections are activated by aversive stimuli (Lammel et al., 2012) and it has been shown that different kinds of aversive experiences increase DA levels within this cortex (Abercrombie et al., 1989; Horvitz, 2000; Bassareo et al., 2002), suggesting that DA signaling in mPFC may play a critical role in the lasting storage of memories for fearful or noxious stimuli. Previously, we showed that maintenance of fear memory is modulated by the VTA through the late posttraining activation of DA D1/D5 receptors in the dorsal hippocampus (Rossato et al., 2009). This activation triggers a late consolidation phase that requires gene expression and protein synthesis, and selectively promotes persistence, but does not affect formation of hippocampus-dependent aversive memories (Bekinschtein et al., 2007; Rossato et al., 2009). However, little is known about the involvement of mPFC in late memory processing and even less regarding its participation in memory storage. We found that activation of D1/D5 receptors in mPFC immediately and late after learning is critical for maintenance of the long-term memory (LTM) trace induced by both hippocampus-dependent and hippocampus-independent aversion-motivated learning tasks, indicating that mPFC regulates memory durability regardless of the aversive properties of the stored information.

## Materials and methods

### Subjects

Experiments were conducted in male Wistar rats (UBA, Argentina and UFRN, Brazil) weighting 220–250 g. Animals were housed five to a cage and kept at a constant temperature of 23°C, with water and food *ad libitum*, under a 12-h light/dark cycle (lights on at 7:00 a.m.). Experimental procedures were performed in accordance with the USA National Institutes of Health Guide for the Care and Use of Laboratory Animals and were approved by the Animal Care and Use Committees of the University of Buenos Aires (CICUAL).

### Surgery

Animals were anesthetized with a mix of ketamine (85 mg/kg) and xylazine (10 mg/kg), and placed on a stereotaxic frame. The skull was exposed and leveled (flat skull, lambda and bregma at the same elevation). 22-G guide cannulas were bilaterally implanted, aimed to the mPFC: AP +3.20 mm/LL ±0.75 mm/DV −3.20 mm or the CA1 region of the dorsal hippocampus: AP −3.90 mm/LL ±3.00 mm/DV −3.00 mm (from Bregma; Paxinos and Watson, 1997) (Figure S1). Cannulas were fixed to the skull with dental acrylic. Immediately after surgery, animals were injected with a single dose of meloxicam (0.2 mg/kg) as analgesic. Animals were allowed to recover from surgery for 5–7 days before any experimental manipulation.

### Inhibitory avoidance task

After recovery from surgery, animals were handled once a day for 2 days and then trained in the inhibitory avoidance task (IA) as described previously (Bekinschtein et al., 2007). Briefly the apparatus was a 50 × 25 × 25 cm acrylic box whose floor was a grid made of 1 mm-caliber steel bars. The left end of the grid was covered with a 7 cm-wide, 5 cm-high wooden platform. For training, animals were placed on the platform and as they stepped down onto the grid received a single 3-s, 0.8 mA scrambled footshock (strong training) or a 3-s, 0.4 mA scrambled footshock (weak training). Rats were tested for retention 2, 7, or 14 days after training, depending on the experiment. All animals were tested only once. In the test sessions the footshock was omitted. The time spent on the platform during this session was taken as an indicator of retention.

### Conditioned taste aversion task

After recovery from surgery, animals were trained in the conditioned taste aversion (CTA) task as described previously (Ballarini et al., 2009). Briefly, animals were deprived of water for 24 h and then habituated to drink water from a graduated tube for 20 min each day for 3 days. In the training session, water was substituted with a 0.1% saccharin (Sigma-Aldrich) solution and 30 min later the animals were injected i.p. with 0.4 M LiCl (Sigma-Aldrich, 7.5 ml/kg) that produces a CTA memory lasting for at least 20 days. During the tests session, water was again replaced by a 0.1% saccharin solution. Training and test sessions lasted 20 min. Rats were tested for retention 3 days (early LTM) or 20 days (remote LTM) after training. All animals were tested only once. Saccharin consumption was calculated as follow: consumption in the test session × 100/consumption in the training session.

### Drug administration

Infusions were delivered through an injector cannula extending 1 mm beyond the tip of the guide cannula. The volume infused was 1 μl per side and the infusion rate was 0.5 μl/min. The injector was left in place for an additional minute after infusion to allow diffusion and to prevent reflux. Doses were as follow: emetine 50 μg/μl, muscimol 0.1 μg/μl, SCH23390 hydrochloride 1.5 μg/μl or SKF38393 hydrochloride 12.5 μg/μl (Sigma Aldrich, St Louis, MO). Drugs were dissolved in sterile 0.9% saline, except for SKF38393 hydrochloride which was dissolved in 10% DMSO. The doses utilized were determined based on previous studies showing the effect of each compound on learning or behavioral performance (Majchrzak and Di Scala, 2000; Lima et al., 2009; Kramar et al., 2014).

### Histology

Cannula placement was verified as previously described (Tomaiuolo et al., 2014). Animals were killed by decapitation at the end of the experiment, brains were fixed in 4% PFA for 2 days and sliced in 100 μm coronal sections to corroborate the injection site (Figure S1). Rats found to have misplaced guide cannula were excluded from behavioral analysis.

### Data analysis

Data were analyzed by unpaired Student's *t*-test or One-Way ANOVA followed by Newman–Keuls multiple comparison tests, as appropriate. IA data are expressed as mean ± SEM of training or test session step-down latency. CTA data are expressed as mean percentage ± SEM relative to the training session.

## Results

**Figure 1:**
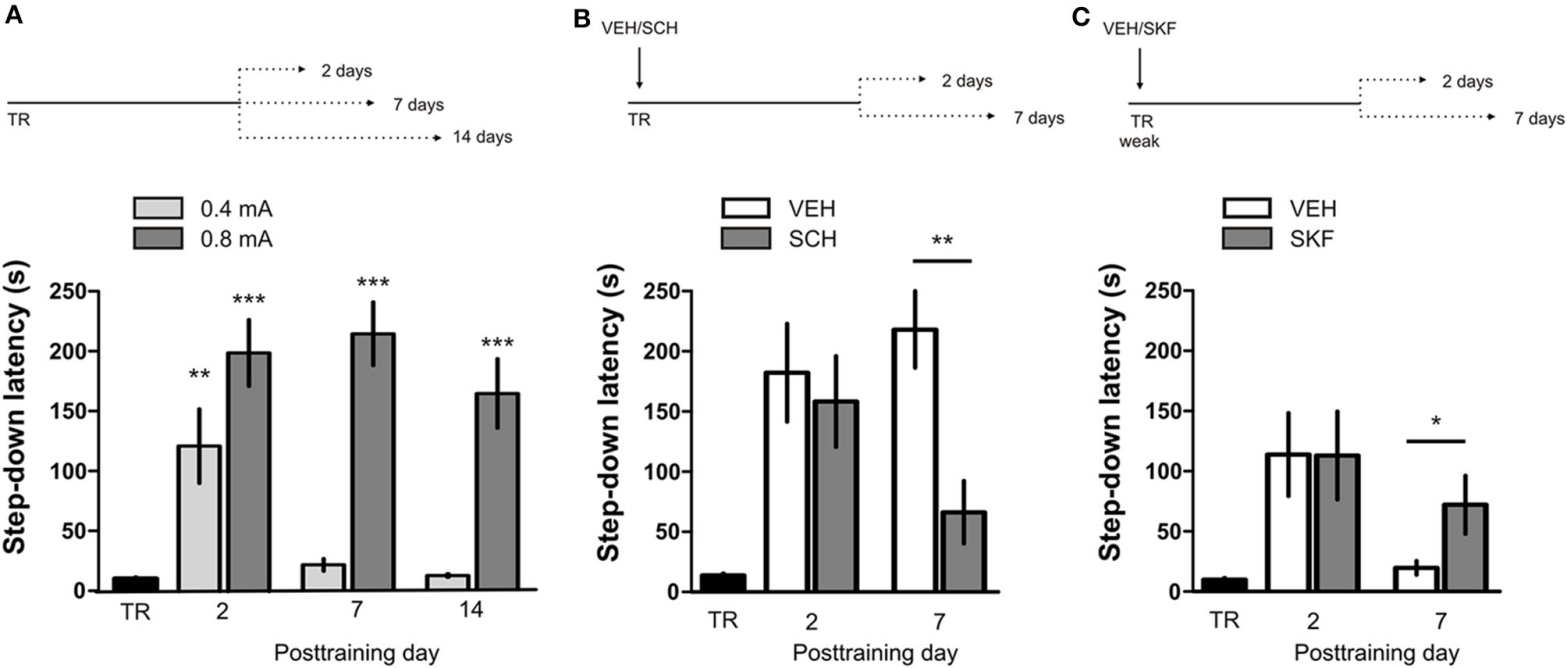
**D1/D5 receptors activity in mPFC immediately after training determines LTM persistence. (A)** Animals were trained in IA using a weak (0.4 mA) or a strong (0.8 mA) footshock as unconditioned stimulus. Memory retention was evaluated 2, 7, or 14 days posttraining. **(B)** Animals were trained in IA using a strong footshock and immediately after that received bilateral intra-mPFC infusions of vehicle (VEH) or SCH23390 (SCH). **(C)** Animals were trained in IA using a weak footshock and immediately after that received bilateral intra-mPFC infusions of VEH or SKF38393 (SKF). Memory retention was evaluated 2 or 7 days after training. ^*^p < 0.05, ^**^p < 0.01, ^***^p < 0.001; TR, training.

**Figure 2:**
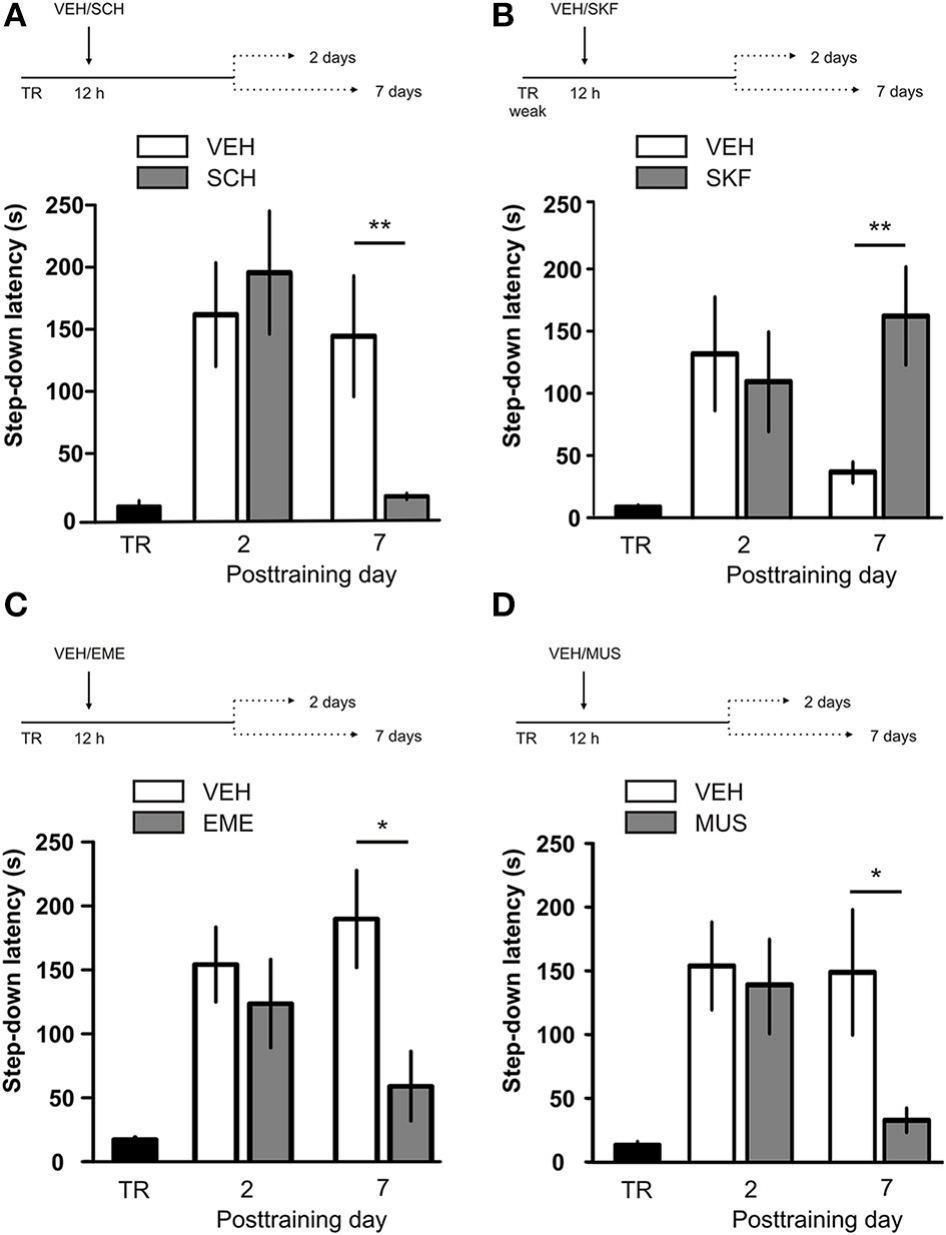
**D1/D5 receptors activity, protein synthesis and neural activity in mPFC 12 h after training determine LTM persistence. (A)** Animals were trained in IA with a strong footshock and 12 h later received bilateral intra-mPFC infusions of vehicle (VEH) or SCH23390 (SCH). **(B)** Animals were trained in IA with a weak footshock and 12 h later received bilateral intra-mPFC infusions of VEH or SKF38393 (SKF). **(C)** Animals were trained in IA with a strong footshock and 12 h later received bilateral intra-mPFC infusions of VEH or emetine (EME). **(D)** Animals were trained in IA with a strong footshock and 12 h later received bilateral intra-mPFC infusions of VEH or muscimol (MUS). Memory retention was evaluated 2 or 7 days after training. ^*^p < 0.05, ^**^p < 0.01; TR, training.

**Figure 3:**
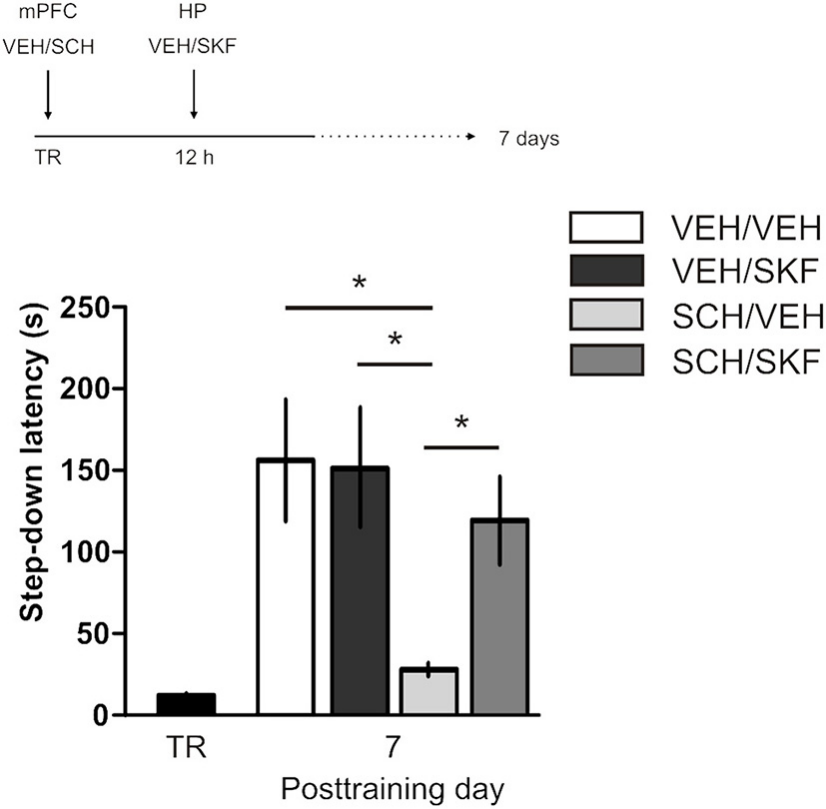
**Late posttraining activation of hippocampal D1/D5 receptors rescues the memory deficit caused by early blockade of D1/D5 receptors in mPFC**. Animals were trained in IA with a strong footshock and immediately after that received bilateral intra-mPFC infusions of vehicle (VEH) or SCH23390 (SCH) plus bilateral intra-CA1 hippocampal infusions of VEH or SKF38393 (SKF) 12 h later. Memory retention was evaluated 7 days after training. ^*^p < 0.05; TR, training.

**Figure 4:**
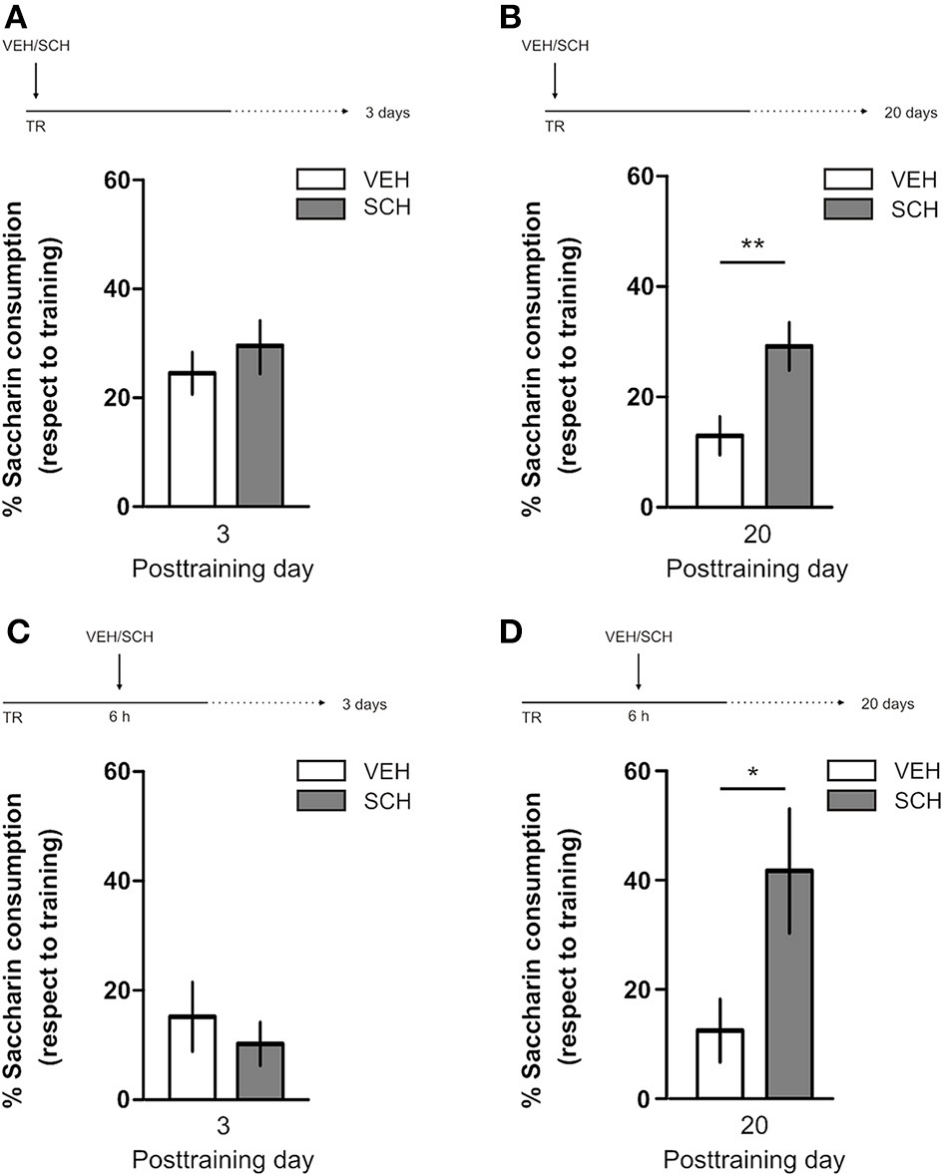
**D1/D5 receptors activity in mPFC early and late after training determines CTA LTM persistence**. Animals were trained in CTA and immediately posttraining received bilateral intra-mPFC infusions of vehicle (VEH) or SCH23390 (SCH). Memory retention was evaluated 3 days **(A)** or 20 days **(B)** after training. Animals were trained in CTA and 6 h after that received bilateral intra-mPFC infusions of VEH or SCH. Memory retention was evaluated 3 days **(C)** or 20 days **(D)** after training. ^*^p < 0.05, ^**^p < 0.01. TR, training.

IA LTM duration can be adjusted by modifying the footshock strength at the moment of training. While a weak footshock (0.4 mA) induces a short-lasting IA LTM enduring no more than 2–3 days, a strong footshock (0.8 mA) produces a persistent aversive memory trace lasting over 14 days [Figure 1A; *F*_(6, 110)_ = 14.19, *p* < 0.0001; TR vs. 0.4 mA 2-days test, ^**^p < 0. 01; TR vs. 0.8 mA 2-, 7-, or 14-days test, ^**^p < 0.001; *n* = 10–20 rats per group]. Given that VTA dopamine neurons signal aversion, saliency and novelty (Lammel et al., 2008, 2011; Bromberg-Martin et al., 2010) and a subset of VTA DA neurons projecting to mPFC are activated by aversive stimuli (Abercrombie et al., 1989; Bassareo et al., 2002; Brischoux et al., 2009; Lammel et al., 2011, 2012), we investigated whether D1/D5 DA receptors in mPFC are involved in IA LTM processing. We performed intra-mPFC infusions of the D1/D5 receptor antagonist SCH23390 (SCH) immediately after strong IA training. SCH impaired retention when memory was assessed 7 days after training, without affecting the 2-day memory expression (Figure 1B, ^**^p < 0.01, *n* = 11–12). Conversely, intra-mPFC infusion of the D1/D5 receptor agonist SKF38393 (SKF) immediately after weak IA training enhanced memory retention 7 days, but not 2 days posttraining (Figure 1C, ^*^p < 0.05, *n* = 12–13). These results indicate that D1/D5 DA receptor signaling in the mPFC is required around training to establish a persistent LTM. Then, we examined the effect of blocking mPFC D1/D5 DA receptors late after IA training on memory maintenance. Intra-mPFC administration of SCH 12 h after strong IA training impaired memory retention 7 days, but not 2 days later (Figure 2A, ^**^p < 0.01, *n* = 7–8). Conversely, SKF specifically increased IA LTM persistence (Figure 2B, ^**^p < 0.01, *n* = 7–8). These results indicate that D1/D5 receptors in the mPFC are required late after training for persistent IA LTM, but not for IA LTM formation. D1/D5 receptors modulate the late protein synthesis-dependent phase of LTP in the hippocampus (Huang and Kandel, 1995; Navakkode et al., 2007). We found that bilateral intra-mPFC infusion of the protein synthesis inhibitor emetine (EME) 12 h after training impaired IA LTM 7 days after training. No effect on retention was observed when LTM was tested 2 days posttraining (Figure 2C, ^*^p < 0.05, *n* = 10–16). Therefore, protein synthesis in the mPFC is required late after training to maintain IA LTM persistence. Late posttraining neural activity in the mPFC is also necessary for the persistence of IA LTM storage since bilateral infusions of the GABA_A_ receptor agonist muscimol (MUS) in this cortex impaired memory retention when tested 7 days, but not 2 days after training (Figure 2D, ^*^p < 0.05, *n* = 6–12). Previously, we demonstrated that persistent LTM depends on late but not early posttraining activation of hippocampal D1/D5 receptors regulated by the VTA (Rossato et al., 2009). After establishing that normal functionality of mPFC dopamine signaling at the moment of training controls the duration of IA LTM, we next investigated the possible interplay between mPFC and the hippocampus to maintain IA memory storage. We found that intra-CA1 infusion of SKF 12 h after strong IA training reversed the amnesic effect of the immediate posttraining intra-mPFC administration of SCH, suggesting that there is a functional link between the early posttraining activation of D1/D5 DA receptors in mPFC and the DA-dependent late consolidation phase in the hippocampus [Figure 3, *F*_(3, 35)_ = 4.113, *p* = 0.0134; *post-hoc* comparisons: VEH/VEH vs. SCH/VEH, ^*^p < 0.05; SCH/VEH vs. SCH/SKF, ^*^p < 0.05; SCH/VEH vs. VEH/SKF, ^*^p < 0.05; VEH/VEH vs. SCH/SKF, VEH/SKF vs. SCH/SKF, or VEH/VEH vs. VEH/SKF, ns; *n* = 8–11 rats per group]. CTA is a rapid and robust model for aversive memory in which rats acquire aversion to a novel taste when this taste is associated with a digestive malaise (Rosenblum et al., 1993; Bermúdez-Rattoni et al., 2004). This learning task requires the functional participation of the insular cortex and the amygdala but not of the hippocampus (Rosenblum et al., 1993; Guzmán-Ramos et al., 2010). CTA memory becomes sensible to protein synthesis inhibition in the insular cortex soon after training and again 5–7 h later (Rosenblum et al., 1993; Berman and Dudai, 2001; Moguel-González et al., 2008; Martínez-Moreno et al., 2011), suggesting that early and late consolidation phases occur in the insular cortex to form CTA LTM. To assess whether mPFC dopaminergic activity is involved in the persistent storage of hippocampus-independent aversive memories, we studied the effect of mPFC D1/D5 receptors blockade on CTA memory maintenance. Intra-mPFC infusion of SCH immediately after training did not affect CTA memory evaluated 3 days later (Figure 4A, *n* = 12–14), but impaired retention when CTA memory was evaluated 20 days posttraining (Figure 4B, ^**^p < 0.01, *n* = 12–13). Similarly, intra-mPFC infusion of SCH 6 h after training did not affect CTA memory evaluated 3 days later (Figure 4C, *n* = 8), but impaired retention when CTA memory was evaluated 20 days posttraining (Figure 4D, ^*^p < 0.05, *n* = 8–10). Therefore, normal maintenance of long-lasting CTA memory requires an early and a late phase of DA signaling in the mPFC. No differences among SCH or vehicle groups were found for baseline water intake or saccharin consumption during CTA training (Table S1).

## Discussion

The main finding of the present study is that mPFC D1/D5 receptors regulate the long-lasting storage of hippocampus-dependent and hippocampus-independent aversive memories. We demonstrated that early posttraining D1/D5 receptor activity in mPFC is necessary and sufficient for memory persistence but not for memory formation (Figures 1B,C). This is consistent with results showing that mPFC D1/D5 receptors are not involved in encoding an olfactory fear conditioning memory (Lauzon et al., 2009). In contrast to our findings, Runyan and Dash described that intra-mPFC administration of SCH before fear conditioning impairs LTM retention (Runyan and Dash, 2004). However, more recent findings suggest that learning reactive fear responses (contextual fear conditioning) engages different brain circuitry than learning active fear responses (inhibitory avoidance task) (Yang and Liang, 2014), suggesting that memory processing could differ between tasks. Also, distinct experimental procedures such as time of drug administration (before vs. after training) or intrinsic characteristics of the tasks (multi-trial vs. single trial) could explain the differences observed. Recently, we demonstrated that protein synthesis in mPFC around training is necessary for IA LTM consolidation (Gonzalez et al., 2013). This finding impedes the analysis of the effects of early posttraining inhibition of protein synthesis in the mPFC on memory persistence. The late consolidation phase involved in memory persistence requires activation of D1/D5 receptors, neural activity and protein synthesis in mPFC late after training (Figure 2), similarly to what happens in the hippocampus (Bekinschtein et al., 2007; Rossato et al., 2009). Based on recent findings (Lima et al., 2013) and those in Figure 3, we suggest that dopaminergic inputs to the hippocampus are a final common point for neocortical influences on IA memory persistence driven by VTA-mPFC connections at the time of training and 12 h thereafter. The acute mPFC manipulations described here specifically affect memory persistence without altering memory formation, indicating that this cortex is part of the circuitry involved in the maintenance of the information over time together with the hippocampus and the VTA, as previously described by our groups (Bekinschtein et al., 2007; Rossato et al., 2009) and confirmed by others (Parfitt et al., 2011, 2012; Werenicz et al., 2012).

The role of the mPFC in memory processing has attracted much attention in recent years. Based on anatomical and behavioral data it has been postulated that the rodent prelimbic cortex and the ventral anterior cingulated cortex are homologous to the dorsolateral prefrontal cortex in primates (Vertes, 2006; Kesner and Churchwell, 2011). From pioneering studies on working memory (Goldman and Rosvold, 1970; Brito and Brito, 1990; Kesner and Churchwell, 2011) to studies on its role in recent and remote memories (Runyan et al., 2004; Frankland and Bontempi, 2005; Zhang et al., 2011), the mPFC appears to fulfill a wide range of mnemonic functions (Euston et al., 2012). Our findings add a new and important function to this list: the mPFC is not only relevant for the active maintenance of information during seconds (working memory), but it is also essential for storing aversive memory traces. Without the activity of the VTA (Rossato et al., 2009) or the dopaminergic activity in the mPFC at the time of training, no long-lasting LTM can be established and only a non-persistent memory lasting a couple of days is formed. It is known that a subpopulation of VTA neurons projecting to mPFC are excited by both rewarding and aversive events, conveying information regarding the motivational salience of the experience, and/or alerting signals triggered by unexpected sensory cues (Bromberg-Martin et al., 2010; Lammel et al., 2011, 2012). How this information is integrated by mPFC neurons to influence the persistence of memory storage is not known. mPFC neurons display periods of burst firing in response to salient stimuli (Burgos-Robles et al., 2007) and burst-like activation of mPFC neurons induces a massive increase in DA neuron activity (Lodge, 2011). Several anatomical and electrophysiological studies demonstrate that mPFC regulates VTA dopaminergic activity by innervating those DA neurons that project back to the mPFC (Overton et al., 1996; Carr and Sesack, 2000; Aston-Jones et al., 2009; Lodge, 2011). In this context, it is interesting to note that intra-VTA infusion of the NMDA receptor antagonist AP5 immediately after IA training elicits a selective impairment of memory persistence without affecting memory formation (Rossato et al., 2009).

We suggest that persistence of fear memory storage depends on functional interactions between the mPFC and VTA. The early activation of mPFC dopaminergic connections, via D1/D5 receptors, is critical for maintenance of the memory trace, while mPFC and hippocampus dopaminergic VTA projections are required late after training for memory persistence. Thus, activation of the VTA-hippocampus dopamine loop (Rossato et al., 2009) seems to be a final common pathway for influences coming from different cortical and subcortical regions (Figure 3 and Lima et al., 2013) to support a late consolidation phase of hippocampus-dependent aversive memories. Also, the effects of early posttraining manipulation of mPFC dopaminergic signaling indicate that this circuit is recruited before the hippocampus in the process to support memory persistence. Besides, we demonstrated that dopamine signaling in mPFC controls the persistence of an aversive no-fear related memory. Long-lasting storage of CTA LTM requires early and late posttraining activation of D1/D5 receptors in mPFC. Supporting the participation of this cortex in CTA memory processing, a recent work has shown that prelimbic activity synchronized with the insular cortex and the amygdala is necessary for CTA learning (Uematsu et al., 2014). It would be interesting to conduct further experiments to study a putative interplay between the mPFC DA signaling and the late consolidation phase described in the insular cortex (Martínez-Moreno et al., 2011). It could be possible that in this case, the insular cortex is the final common pathway to support the persistent storage of this hippocampus-independent aversive memory.

In conclusion, the present results show that early and late posttraining D1/D5 dopaminergic neurotransmission in the mPFC plays a key role in the persistent storage of different types of aversive memories. This is independent of the dynamics of CTA and IA memory consolidation and of the participation or lack thereof of the hippocampus in this process. Since catecholamine deregulation in the prefrontal cortex has been related to many psychiatric disorders (Arnsten, 2007; Gamo and Arnsten, 2011), we propose that disturbances in the D1-like receptor circuitry may be underlying the abnormal persistent storage of aversive memories, such as that observed in phobias or PTSD. Further research is needed to fully understand this phenomenon and use it as a potential therapeutic target to control the aberrant overexpression of aversive memories.

### Conflict of interest statement

The authors declare that the research was conducted in the absence of any commercial or financial relationships that could be construed as a potential conflict of interest.

## Supplementary material

Figure S1 **Histological analysis of cannula placement**. Top: Representative microphotograph showing the position of guide cannulas in mPFC **(A)** and the CA1 region of the dorsal hippocampus **(B)**. Scale bar 1 mm. Bottom: Schematic illustration of the injection sites in the intended areas: mPFC, rat brain section at rostrocaudal plane +3.20 **(A)** and CA1 region of the dorsal hippocampus, rat brain section at rostrocaudal plane −3.80 **(B)** from Bregma taken from the atlas of Paxinos and Watson (1997).
