# Supplementary figures and images for: Medial prefrontal cortex dopamine controls the persistent storage of aversive memories
Source: Front Behav Neurosci. 2014 Nov 26;8:408. doi: 10.3389/fnbeh.2014.00408 (PMC4246460; doi:10.3389/fnbeh.2014.00408)

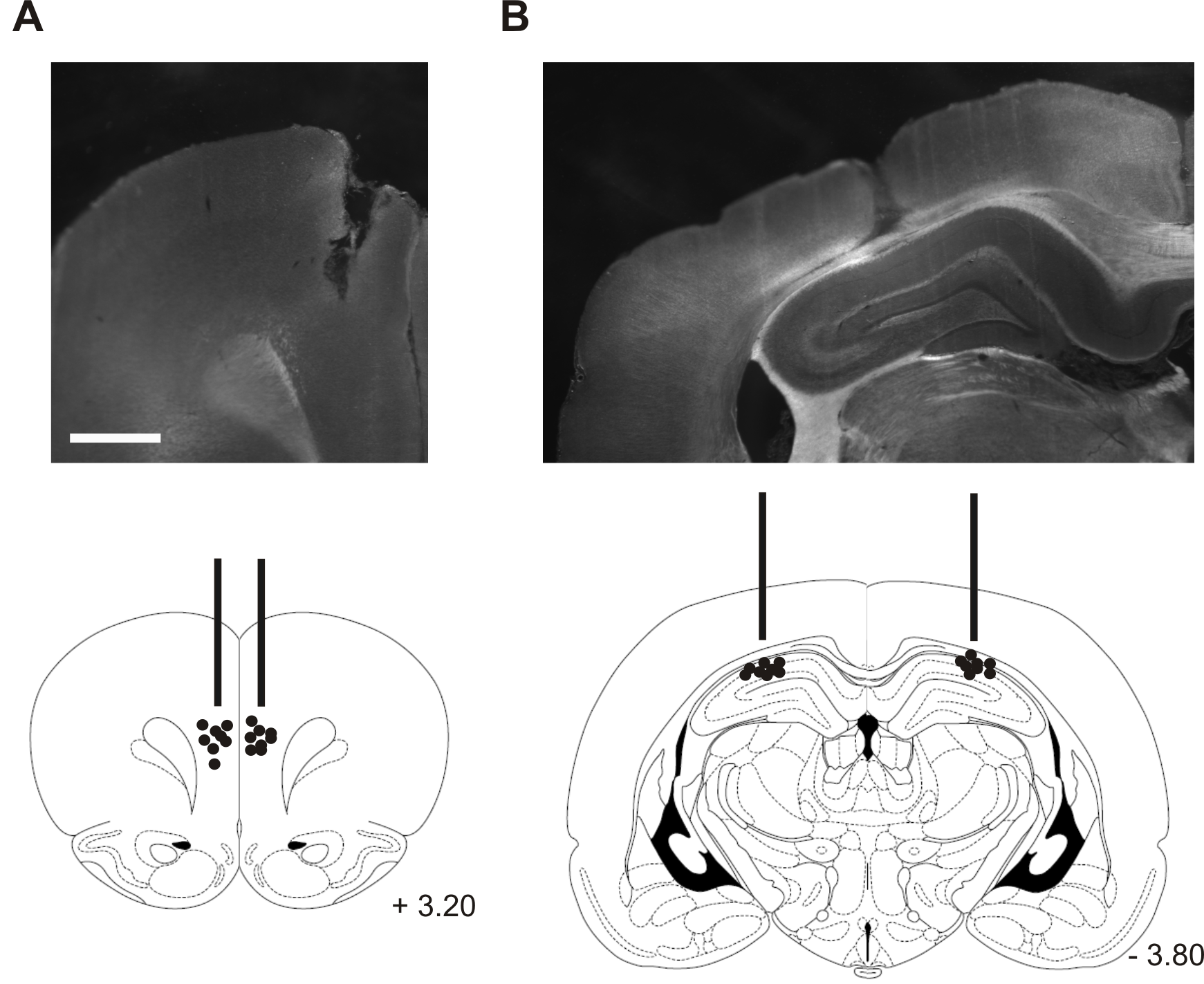

Supplement: Figure S1 — Histological analysis of cannula placement. Top: Representative microphotograph showing the position of guide cannulas in mPFC (A) and the CA1 region of the dorsal hippocampus (B). Scale bar 1 mm. Bottom: Schematic illustration of the injection sites in the intended areas: mPFC, rat brain section at rostrocaudal plane +3.20 (A) and CA1 region of the dorsal hippocampus, rat brain section at rostrocaudal plane −3.80 (B) from Bregma taken from the atlas of Paxinos and Watson (1997). [file Image1.TIF]
