# Supplementary material for: Medial prefrontal cortex dopamine controls the persistent storage of aversive memories
Source: Front Behav Neurosci. 2014 Nov 26;8:408. doi: 10.3389/fnbeh.2014.00408 (PMC4246460; doi:10.3389/fnbeh.2014.00408)
Supplement: Supplementary file 2 [file Table1.DOC]

**Table S1.** Average mean (±SEM) of baseline water intake before training or saccharin intake during training (in ml).

|  | VEH 0h Test-3d | SCH 0h Test-3d | VEH 0h Test-20d | SCH 0h Test-20d | VEH 6h Test-3d | SCH 6h Test-3d | VEH 6h Test-20d | SCH 6h Test-20d |
| --- | --- | --- | --- | --- | --- | --- | --- | --- |
| Water intake (ml) | 13.14 ± 3.8 | 9.63 ± 2.65 | 10.79 ± 1.67 | 10.79 ± 1.25 | 11.38 ± 2.11 | 12.00 ± 1.48 | 10.86 ± 1.55 | 10.50 ± 2.05 |
| Saccharin intake (ml) | 11.82 ± 2.12 | 8.59 ± 1.81 | 11.33 ± 1.82 | 10.13± 1.93 | 9.88± 2.82 | 10.81 ± 2.91 | 10.11 ± 1.89 | 10.19 ± 1.66 |
